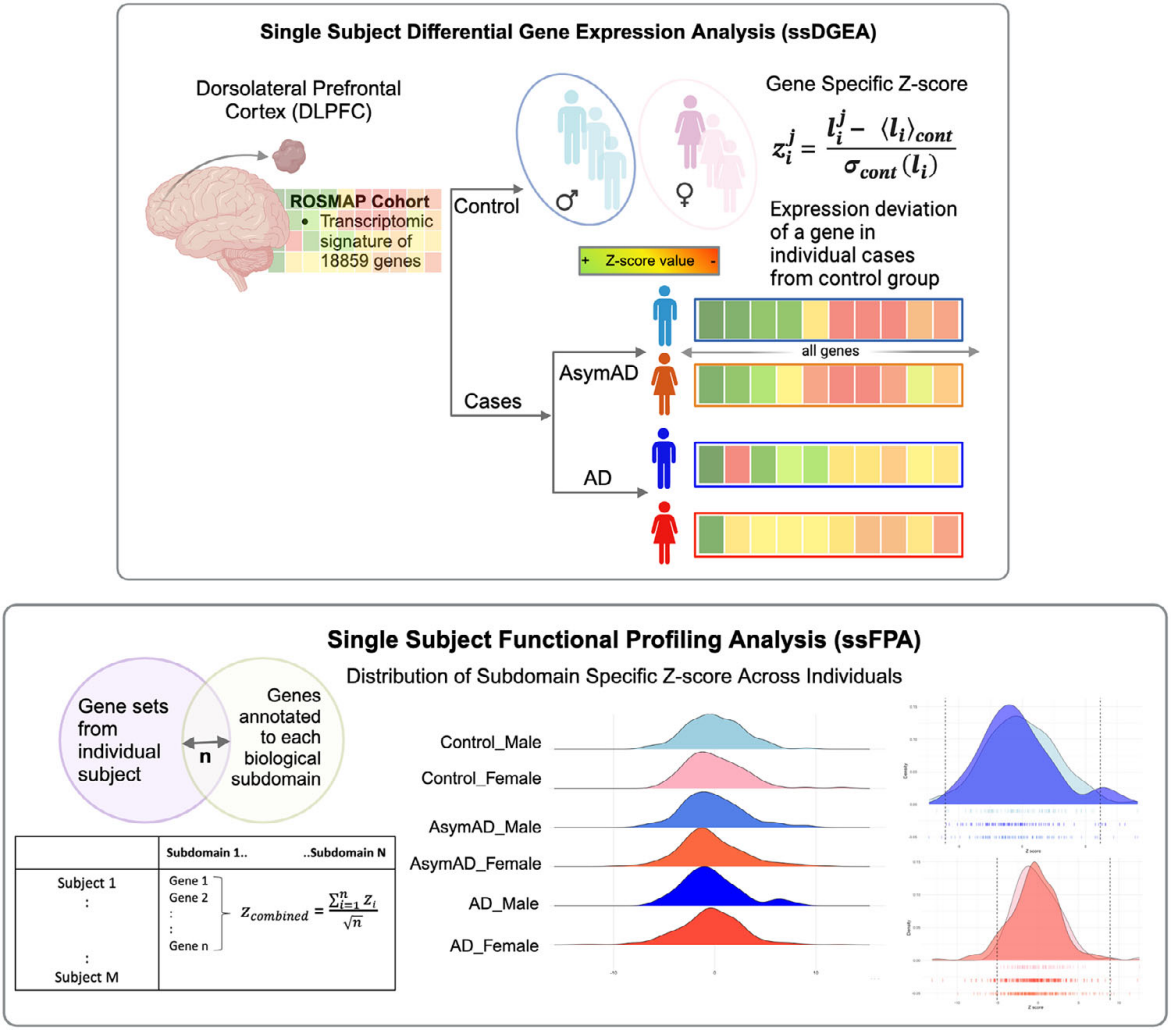# Harnessing Individual Omics Signatures for Precision Medicine in Alzheimer's Disease

**DOI:** 10.1002/alz70855_107191

**Published:** 2025-12-25

**Authors:** Avijit Podder, Yi Juin Liew, Ravi S Pandey, Gregory A. Cary, Gregory W Carter, Asli Uyar

**Affiliations:** ^1^ The Jackson Laboratory for Genomic Medicine, Farmington, CT, USA; ^2^ The Jackson Laboratory, Bar Harbor, ME, USA

## Abstract

**Background:**

Alzheimer's disease (AD) exhibits substantial molecular heterogeneity across individuals; however, conventional cohort‐based case vs. control omics analyses often mask individual‐level variability in molecular disease mechanisms. Emerging single‐subject omics approaches enable a more granular understanding of AD by capturing pathway‐specific disruptions at the patient level. Leveraging these methods can improve disease stratification and inform personalized therapeutic strategies.

**Methods:**

We analyzed RNA‐Seq transcriptomics and proteomics data from post‐mortem dorsolateral prefrontal cortex (DLPFC) in the ROSMAP cohort (913 individuals: 377 AD, 345 asymptomatic AD (asymAD), 191 controls). Individual‐based gene deregulation was assessed via z‐score distribution relative to sex‐matched controls. We conducted single‐subject KEGG pathway and AD biodomain functional enrichment analysis and utilized an unsupervised graph‐based clustering approach to stratify patients into subgroups based on similarity of pathway alterations. Correlations between clinical metrics and omics signatures were evaluated at individual and subgroup levels.

**Results:**

Our single‐subject approach revealed individualized, sex‐dependent functional dysregulation in AD. In the transcriptomic profile, the peroxisome pathway was primarily downregulated in males (15% males vs. 3% females), while the MAPK signaling pathway was upregulated in females (17% females vs. 3% males). Endolysosome‐related processes were predominantly upregulated in male AD individuals, whereas myelination‐related subdomains were more pronounced in females. Apoptosis and epigenetic‐related biodomains distinguished AD from asymAD, suggesting links to disease progression. Clustering analyses stratified individuals into molecular subtypes defined by cell cycle, DNA repair, synapse, and endolysosome‐related subdomains, capturing heterogeneity beyond traditional diagnoses. Amyloid burden is correlated with oxidative stress in AD but not asymAD. Proteomics analyses aligned with transcriptomics findings, highlighting epigenetic regulation; and additional proteomics‐specific modules indicative of post‐transcriptional mechanisms.

**Conclusions:**

Our findings underscore the molecular heterogeneity of AD, revealing individual and sex‐dependent functional dysregulation better captured through single‐subject analyses than cohort‐based approaches. Clustering identified biologically distinct subgroups, some correlating with cognitive measures, providing potential biomarkers for refined disease stratification. These insights offer a framework for precision medicine in AD, guiding targeted therapeutic strategies based on individualized omics molecular signatures.